# Clinical outcomes associated with long-term exposure to airborne particulate pollution in kidney transplant recipients

**DOI:** 10.1186/s12940-021-00741-y

**Published:** 2021-05-15

**Authors:** Yong Chul Kim, Ejin Kim, Jiyun Jung, Jae Yoon Park, Hajeong Lee, Dong Ki Kim, Yon Su Kim, Chun Soo Lim, Jung Pyo Lee, Ho Kim

**Affiliations:** 1 Department of Internal Medicine, Seoul National University Hospital, Seoul, Republic of Korea; 2 Institute of Health and Environment, Graduate School of Public Health, Seoul National University, Seoul, Republic of Korea; 3 Department of Public Health Science, Institute of Sustainable Development, Institute of Health and Environment, Graduate School of Public Health, Seoul National University, Room 708, Building 220, Gwanak-Ro Gwanak-Gu, Seoul, 08826 Republic of Korea; 4 Department of Internal Medicine, Dongguk University Ilsan Hospital, Gyeonggi-do, Republic of Korea; 5 Department of Internal Medicine, Seoul National University College of Medicine, 20 Boramae-ro 5-gil, Dongjak-gu, Seoul, 07061 Republic of Korea; 6 Kidney Research Institute, Seoul National University Hospital, Seoul, Korea; 7 Department of Medical Science, Seoul National University College of Medicine, Seoul, Korea; 8 Department of Internal Medicine, Seoul National University Boramae Medical Center, 20 Boramae-ro 5-gil, Dongjak-gu, Seoul 07061 Seoul, Republic of Korea

**Keywords:** Long-term PM10 exposure, Kidney transplant recipients, Outcomes

## Abstract

**Background:**

Researchers have yet to investigate the specific association between 10-μm particulate matter (PM10) levels and the risk of graft failure, kidney disease, or the functional decline of transplanted kidneys, in kidney transplant recipients (KTRs). Furthermore, we know very little about the association between PM10 levels and the development of allograft rejection in transplanted kidneys. Identification of air pollution as a potential contributor to kidney disease could help reduce future disease burden, stimulate policy discussions on the importance of reducing air pollution with respect to health and disease, and increase public awareness of the hazards of air pollution. We aimed to evaluate the relationship of PM10 with the risk of graft failure, mortality, and decline of graft function in KTRs.

**Methods:**

Air pollutant data were obtained from the Korean National Institute of Environmental Research. We then investigated potential associations between these data and the clinical outcomes of 1532 KTRs who underwent kidney transplantation in a tertiary hospital between 2001 and 2015. Survival models were used to evaluate the association between PM10 concentrations and the risk of death-censored graft failure (DCGF), all-cause mortality, and biopsy-proven rejection (BPR), over a median follow-up period of 6.31 years.

**Results:**

The annual mean PM10 exposure after kidney transplantation was 27.1 ± 8.0 μg/m^3^. Based on 1-year baseline exposure, 1 μg/m^3^ increase in PM10 concentration was associated with an increased risk of DCGF (hazard ratio (HR): 1.049; 95% confidence interval (CI): 1.014–1.084) and BPR (HR: 1.053; 95% CI: 1.042–1.063). Fully adjusted models showed that all-cause mortality was significantly associated with 1-year average PM10 concentrations (HR, 1.09; 95% CI, 1.043 to 1.140).

**Conclusions:**

Long-term PM10 exposure is significantly associated with BPR, DCGF, and all-cause mortality in KTRs.

**Supplementary Information:**

The online version contains supplementary material available at 10.1186/s12940-021-00741-y.

## Background

Exposure to outdoor air pollution is a leading cause of global disease burden and accounts for over four million deaths each year [1]. Increased concentrations of daily fine particulates that are 10 μm in aerodynamic diameter (PM10) are associated with an increased risk of cardiovascular disease [2, 3], stroke [4], heart failure [5, 6], death [7], and reduced life expectancy, as well as a host of other adverse health outcomes [1].

In a large cohort of veterans in the United States [8, 9], higher amounts of fine particulate matter were associated with an increased risk of incident chronic kidney disease (CKD), a reduction in estimated glomerular filtration rate (eGFR), and end-stage renal disease (ESRD). Experimental laboratory evidence also indicates that long-term exposure to micro-particles leads to disturbances in renal hemodynamics, thus promoting oxidative stress and systemic inflammation in the renal tissue; these factors exacerbates acute kidney injury and further promotes chronic renal injury in murine models [10, 11].

Previous studies have focused only on evaluating the association between PM10 and the outcomes of kidney diseases in native kidneys. However, researchers have yet to investigate the specific association between PM10 levels and the risk of graft failure, kidney disease, or the functional decline of transplanted kidneys, in kidney transplant recipients (KTRs). Furthermore, we know very little about the association between PM10 levels and the development of allograft rejection in transplanted kidneys. The identification of air pollution as a potential contributor to kidney disease could help reduce future disease burden, stimulate policy discussions on the importance of reducing air pollution with respect to health and disease, and increase public awareness relating to the hazardous nature of air pollution.

In the present study, we developed a longitudinal national cohort of KTRs and investigated the relationship between PM10 levels and the risk of biopsy-proven rejections (BPR), death-censored graft failure (DCGF), and mortality.

## Methods

### Study population

This was a retrospective cohort study involving data generated between January 2001 and December 2015 at the Seoul National University Hospital and Seoul National University Boramae Medical Center. Initially, a total of 1531 adult participants (> 18 years) were included. However, a number of patients were then excluded for the following reasons: (1) multiple organ transplantation (*n* = 61) and double kidney transplantation (*n* = 85); (2) graft failure within 3 months of transplantation (*n* = 7), and (3) mortality within 3 months of transplantation (*n* = 16). Only the participants whose data included a zip code were included in the final analysis.

We also collected a range of baseline information, including recipient/donor age, gender, the relationship between donor and recipient, smoking status, body mass index (BMI), the incidence of diabetes and hypertension, chronic glomerulonephritis as a cause of ESRD, the duration of renal replacement therapy (RRT) prior to transplantation, and the type of previous RRT. We also collated data relating to a range of immunological factors, including preemptive transplantation (defined by the absence of dialysis prior to transplantation), donor status (living or deceased), ABO incompatibility, and a number of HLA-A, −B, and -DR mismatches.

### Air pollution data

Across the South Korea, there are eleven different types of monitoring sites operated by the Ministry of Environment and local governments. These monitoring sites are situated across 584 locations in 114 cities and counties. This study used data arising from 373 monitoring stations situated in both urban and roadside positions.

PM10 concentration data for 2001 and 2015 were provided by the Korean National Institute of Environmental Research (https://www.airkorea.or.kr/web). We collated hourly PM10 data from each of our 373 national monitoring sites. In addition, we used daily mean PM10 concentrations for individual study populations according to the respective district of each enrolled patient, as determined by the residential address stated on medical records.

In this study, we were interested in the effects of long-term exposure. To examine the effect of the amount of exposure at the time of outcome, rather than at the time of kidney transplantation, the exposure concentration was varied from 1 to 5 years before the clinical outcomes. We analyzed the effects of exposure by applying the average concentration values for each of these periods.

### Laboratory measurements and study outcomes

Graft function was assessed by determining serum creatinine levels after transplantation. The eGFR was calculated using the MDRD GFR equation [12]. Graft failure was defined as an irreversible loss of graft function with the need to restart dialysis or to undergo re-transplantation. DCGF data were also analyzed. Surveillance biopsies were routinely performed 1–2 weeks after transplantation in almost all patients. Additional biopsies were performed when renal graft function was impaired, or when there was a considerable reduction in urine output. Rejection was determined according to the diagnostic criteria proposed at the 2007 Banff Conference [13], while BPRs involved acute T-cell mediated rejection (TCMR) and acute antibody-mediated rejection (ABMR), and chronic rejections.

### Statistical analysis

Demographic and clinical characteristics of the overall cohort are presented as frequencies (percentages) for categorical variables and as means (with standard deviation, SD) for continuous variables.

To investigate the association between the clinical outcomes of transplantation patients and PM10 concentration, we calculated Kaplan-Meier estimates of survivor functions and compared survival curves between groups of patients according to a pre-determined cut-off value, which was defined as the median annual mean PM10 concentration.

Next, the median PM10 concentration was used to divide PM10 data into two groups, and a log rank test was used to test the null hypothesis. We then used the Cox proportional hazards survival model to investigate the association between the 1-year mean PM10 concentrations (for the 5-year study period) before the events occurred, along with the individual and clinical outcomes. Finally, we adjusted our data to take account of covariates. As the definition of exposure was dependent on the annual mean obtained from an air monitoring station in each district, we also collated data from the year prior to the event. Additionally, subgroup analysis was performed for two groups of TCMR and ABMR according to the mechanism of occurrence of the biopsy-proven rejection event, one of the outcomes.

To explore whether this association was non-linear or linear, we considered a non-linear relationship via smoothing splines. Splines are mathematical constructs made of fragments of polynomial functions that are stitched together to form a smooth curve. In order to identify a smoothing spline, we used the penalized partial likelihood method; this is known to be an effective method for survival analysis.

We used models that incorporated subject-specific random effects to account for unmeasured subject characteristics that influenced the hazard of the occurrence of the outcome. In our research, these models were then extended to models that incorporated cluster-specific random effects to account for within-cluster homogeneity in outcomes. Moreover, we investigated whether different concentrations of PM10 affected the health of kidney transplant patients using the two-pollutant model of PM10 with adjustment for SO2, CO, NO2, and O3 and whether these effects differed by regions. All statistical analyses were performed using R (3.6.0) and SAS version 9.4.

## Results

**Fig. 1:**
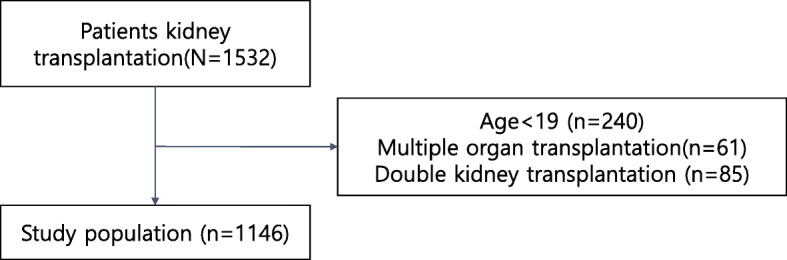
Flow diagram showing patient enrollment and application of exclusion criteria

**Fig. 2:**
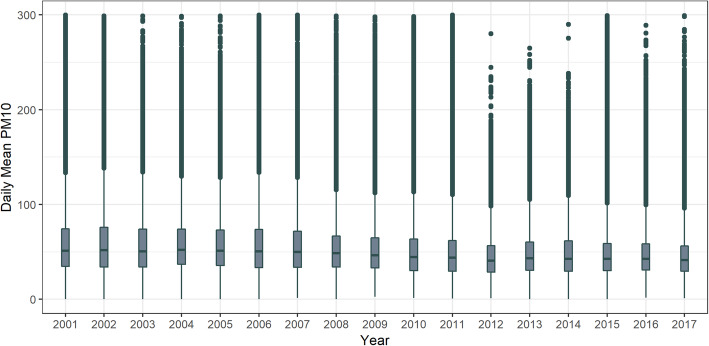
Distribution of baseline PM10 exposure by year. Boxes display the 25-75th percentiles (interquartile range: IQR); the center line represents the median concentration. Whiskers indicate the most extreme data within 3 IQRs of the box, while circles indicate outlying values

Initially, we recruited a total of 1532 individuals. However, we then excluded 240 patients aged < 19 years, 61 patients who underwent multiple organ transplantation, and 85 patients who underwent kidney transplantation more than twice. Consequently, our final analysis featured 1146 eligible KTRs (Fig. 1). The demographic characteristics of the final study participants are presented in Table 1. The mean age of the recipients at the time of kidney transplantation was 45.0 ± 12.5 years and that of the donors was 42.6 ± 13.0 years. The majority of the study population were males (60.12%), and 11.61% were smokers. Overall, 91.97% of our patients were hypertensive and 34.12% had diabetes mellitus. When measured 6 months after transplantation, the eGFR was 56.0 ± 17.5 ml/min/1.73 m^2^. The incidence of preemptive kidney transplantation was 29.8%, while that of ABO incompatible kidney transplantation was 5.9%. The geographic locations of the participants are shown in Figure S1. Figure 2 shows the annual mean PM10 concentrations for each year during the entire study period. The overall mean PM10 concentration was 52.68 ± 30.49 μg/m^3^. The mean PM10 exposure showed a slight increase compared to the overall mean PM10 concentration between 2001 and 2009 (yearly mean PM10 exposure was 60.22, 59.19, 58.05, 58.85, 57.83, 55.9, 56.8, 54.22, and 53.1 μg/m^3^, respectively), but then a slight decrease from 2010 to 2015 (yearly mean PM10 exposure was 51.11, 49.84, 44.96, 48.89, 48.94, and 47.63 μg/m^3^, respectively).

**Table 1:** Demographic and baseline characteristics of the overall study population

Variables	Category	Median		Total *N* = 1146	%	Mean ± SD
	Concentration	Low[26.26 ~ 53.00)	High[53.00 ~ 120.19)			
	N (%)	663 (57.85)	483 (42.15)			
**Recipient characteristics**						
Age at transplantation, years		45.12 ± 12.92	44.92 ± 11.86			45.0 ± 12.5
Sex	male	405 (35.34)	284 (24.78)	689	60.12	
	female	258 (22.51)	199 (17.36)	457	39.88	
Smoking	yes	66 (5.76)	67 (5.85)	133	11.61	
	no	597 (52.09)	416 (36.30)	1013	88.39	
Body mass index, kg/m^2^		22.74 ± 3.35	22.56 ± 3.25			26.2 ± 70.5
Hypertension	yes	588 (51.31)	466 (40.66)	1054	91.97	
	no	75 (6.54)	17 (1.48)	92	8.03	
Diabetes mellitus	yes	229 (19.98)	162 (14.14)	391	34.12	
	no	434 (37.87)	321 (28.01)	755	65.88	
Preemptive	yes	208 (18.15)	134 (11.69)	342	29.84	
	no	455 (39.70)	349 (30.45)	804	70.16	
RRT duration before KT, months		36.93 ± 45.44	39.78 ± 48.28			38.13 ± 46.65
Phosphorus, mg/dl		5.11 ± 6.16	4.91 ± 1.62			5.02 ± 4.82
Albumin, g/dl		5.27 ± 38.66	3.90 ± 0.61			4.69 ± 29.40
eGFR at 6 months, ml/min/1.73m^2^		55.30 ± 18.45	57.06 ± 16.02			56.0 ± 17.5
Diseases causing ESRD	DM	119 (10.38)	70 (6.11)	189	16.49	
	HTN	29 (2.53)	19 (1.66)	48	4.19	
	GN	250 (21.82)	172 (15.01)	422	36.82	
	others	81 (7.07)	73 (6.37)	154	12.43	
	unknown	184 (16.06)	149 (13.00)	333	29.06	
HLA mismatch		2.98 ± 1.76	3.01 ± 1.60			2.99 ± 1.69
**Donor characteristics**						
Donor age, years		43.1 ± 13.14	41.80 ± 12.70			42.6 ± 13.0
Donor sex	male	368 (32.11)	283 (24.69)	651	56.81	
	female	295 (25.74)	200 (17.45)	495	43.19	
Donor type	Living	452 (39.44)	325 (28.36)	777	67.8	
	Deceased	211 (18.41)	158 (13.79)	369	32.2	
ABO	compatible	621 (53.40)	466 (40.66)	1078	94.07	
	incompatible	51 (4.45)	17 (1.48)	68	5.93	

Data are presented as means with standard deviation unless otherwise indicated. Covariates are given as measured at the time of kidney transplantation. *RRT* renal replacement therapy, *KT* kidney transplantation, *eGFR* estimated glomerular filtration rate, *ESRD* end stage renal disease, *DM* diabetes mellitus, *HTN* hypertension, *GN* glomerulonephritis, *HLA* human leukocyte antigen

**Fig. 3:**
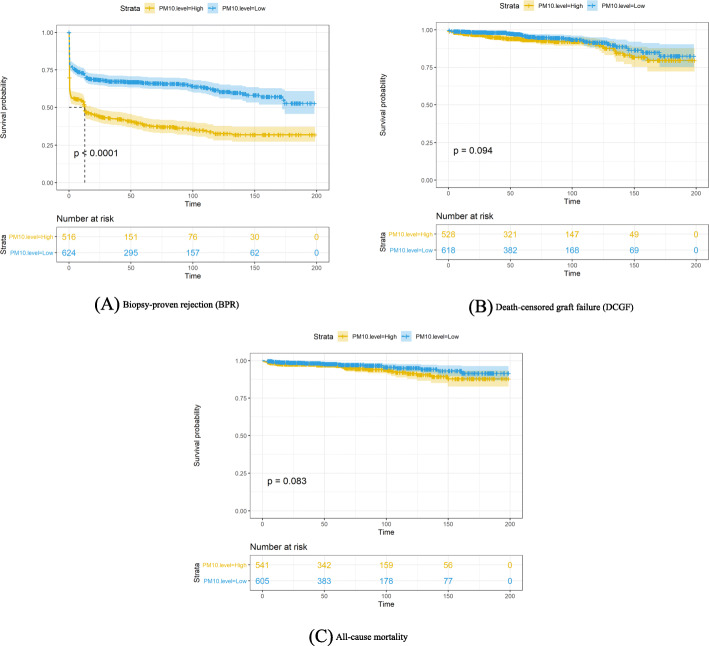
Survival curves by median value of the annual mean PM10 concentration. (**a**) Biopsy-proved rejection (BPR), (**b**) Death-censored graft failure (DCGF), and (**c**) All-cause mortality. Note: The units for the X-axis “Time” of the Kaplan meier curve are months

Mean follow-up time was 6.3 ± 4.2 years and a total of 51 deaths (4.5%), 78 cases of DCGF (6.8%), and 549 cases of BPR (47.9%) were recorded. The Kaplan-Meier survival curves for BPR, DCGF, and mortality were significantly worse in the group exposed to higher concentrations of PM10 than in the group exposed to lower concentrations (Fig. 3).

**Fig. 4:**
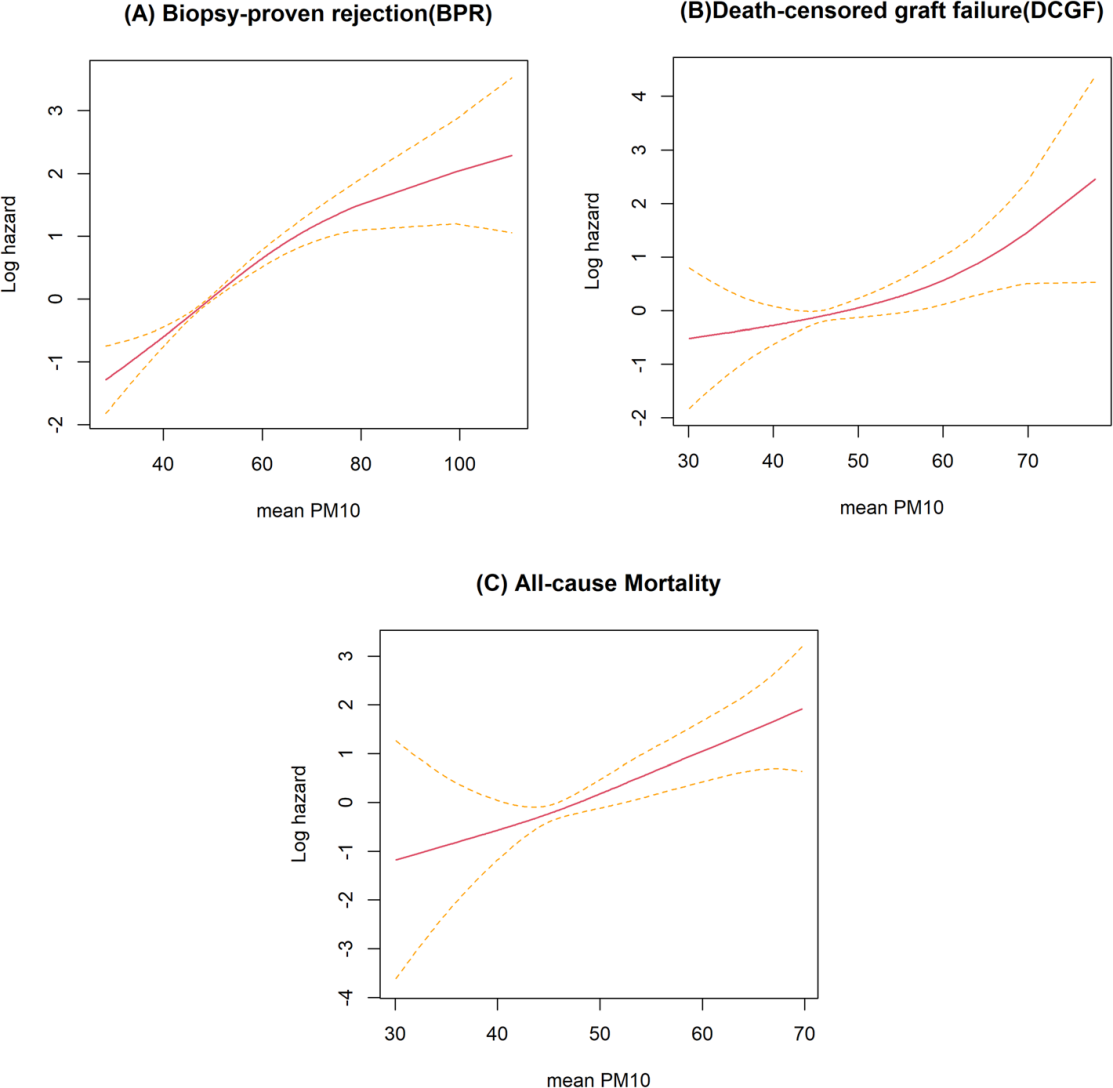
The risk of renal outcomes according to PM_10_ concentrations. Models were adjusted for age, sex, diabetes mellitus, hypertension, eGFR 6 months after surgery, BMI, HLA Ag Mismatch, the cause of ESRD, albumin, phosphorus, preemptive kidney transplantation, donor age, donor sex, and donor type (deceased). **a** Risk of incident biopsy-proven rejections (BPR) (P for nonlinearity = 0.000032). **b** Risk of incident death-censored graft failure (DCGF) (P for nonlinearity = 0.07). **c** Risk of all–cause mortality (P for nonlinearity = 0.4)

Overall, our data indicated that a 1 μg/m^3^ increase in PM10 concentration was associated with an increased risk of BPR (hazard ratio [HR]: 1.053; 95% confidence interval [CI]: 1.042–1.063), graft failure (HR: 1.049; 95% CI: 1.014–1.084), and mortality (HR: 1.090; 95% CI: 1.043–1.140) in our analyses considering exposure over a 1-year period with regards to prospective clinical outcome (Table 2). In addition, we performed analyses of various PM10 exposure values, from 2-year to 5-years (S1-S3 Tables). According to the results of this sensitivity analysis, the effect of PM10 on the clinical outcome of kidney transplant patients consistently showed a significant effect according to the concentration value of PM10 over various periods. Spline analyses suggested a linear relationship between PM10 concentration and the risk of BPR (*P*-value for nonlinearity < 0.031), DCGF (*P*-value for nonlinearity = 0.2), and all-cause mortality (*P*-value for nonlinearity = 0.7; Fig. 4). The slope of the mortality plot showed a linear trend. For BPR, we observed a significant increasing trend in nonlinearity with a slight flat turn after the 1-year moving average PM10 concentration of 70 μg/m^3^. However, only a limited amount of data was acquired at these higher concentrations, as reflected by the wide CI; this confirms our observation that a departure from linearity cannot be established.

**Table 2:** Risk of outcomes in PM _10_ concentration for average of 1 year from events day

	BPR				DCGF				All-cause mortality			
Variables	HR	95% CI		P Value	HR	95% CI		*P* Value	HR	95% CI		*P* Value
Age at transplantation	1	0.991	1.009	0.9997	0.974	0.948	1.002	0.0647	**1.071**	**1.033**	**1.109**	**0.0001**
Smoking	1.168	0.880	1.549	0.2828	1.091	0.507	2.350	0.8235	0.457	0.129	1.612	0.2233
Body mass index	1	0.999	1.001	0.6925	0.928	0.844	1.022	0.1279	0.905	0.801	1.022	0.1087
Hypertension	0.905	0.645	1.269	0.5629	1.198	0.274	5.245	0.8108	0.926	0.116	7.369	0.9422
Diabetes mellitus	0.872	0.675	1.127	0.2948	0.895	0.420	1.907	0.7731	1.167	0.502	2.710	0.72
Sex (male)	1.083	0.885	1.324	0.4382	**2.232**	**1.213**	**4.107**	**0.0098**	1.772	0.834	3.767	0.1368
HLA mismatch	**1.213**	**1.145**	**1.285**	**<.0001**	**1.182**	**1.006**	**1.389**	**0.0419**	0.995	0.805	1.230	0.9628
Cause of ESRD (DM)	1.137	0.808	1.601	0.4602	2.404	0.958	6.035	0.0618	1.381	0.515	3.704	0.521
Cause of ESRD (HTN)	0.986	0.607	1.601	0.9532	1.663	0.471	5.868	0.4291	1.367	0.375	4.983	0.6358
Cause of ESRD (GN)	0.95	0.762	1.185	0.6504	0.682	0.374	1.243	0.2113	0.603	0.256	1.419	0.2466
Cause of ESRD (others)	0.981	0.683	1.409	0.9187				^a^				^a^
Phosphorus	1.047	0.989	1.108	0.1147	**1.252**	**1.089**	**1.440**	**0.0016**	1.2	0.995	1.447	0.0564
Albumin	0.885	0.781	1.002	0.0543	**0.444**	**0.323**	**0.611**	**<.0001**	0.669	0.440	1.015	0.0589
eGFR at 6 months	**0.987**	**0.981**	**0.994**	**<.0001**	**0.965**	**0.946**	**0.984**	**0.0003**	0.999	0.977	1.022	0.9439
preemptive	0.895	0.706	1.134	0.3579	0.815	0.445	1.494	0.509	0.992	0.438	2.247	0.9855
Annual mean PM_10_	**1.053**	**1.042**	**1.063**	**<.0001**	**1.049**	**1.014**	**1.084**	**0.0055**	**1.09**	**1.043**	**1.140**	**0.0001**
Donor age	**1.012**	**1.004**	**1.020**	**0.0036**	0.993	0.971	1.016	0.5561	1.005	0.978	1.033	0.7062
Donor sex (male)	1.146	0.944	1.392	0.1693	1.066	0.633	1.795	0.81	1.539	0.761	3.110	0.2298
Donor type (Deceased)	**1.355**	**1.074**	**1.709**	**0.0105**	1.724	0.884	3.361	0.1098	1.907	0.881	4.129	0.1015
ABO-incompatible	**1.935**	**1.330**	**2.816**	**0.0006**	1.897	0.549	6.558	0.3116	1.205	0.151	9.642	0.8602

Data are presented as mean and standard deviation unless otherwise indicated. Covariates are given as measured at the time of kidney transplantation. *BPR* biopsy-proven rejection, *DCGF* death-censored graft failure, *HLA* human leukocyte antigen, *ESRD* end stage renal disease, *DM* diabetes mellitus, *HTN* hypertension, *GN* glomerulonephritis, *eGFR* estimated glomerular filtration rate. The multivariable model was adjusted for age, smoking, BMI, hypertension, DM, sex, HLA mismatch, cause of ESRD, serum phosphorus, albumin, eGFR at 6 months, preemptive, annual mean PM_10_, donor age, sex, donor type, and ABO-incompatible

^a^These are unestimable due to the lack of the events

The results for the gamma frailty model is reported in Table 3. The estimate of the within-cluster correlation of outcomes and the variance of the frailty distribution for clinical outcomes of BPR, DCGF, and all-cause mortality was 0.05304, 0.0001, and 0.03079, respectively. Thus, the within-cluster correlation of survival times was marginally greater than 0.007, 0.012, and 0.186. After adjustment for air pollutants, the associations between PM10 and BPR and between DCGF and all-cause mortality were significant for all outcomes. The estimates of the hazard ratio for BPR with one unit increase in PM10 concentration decreased significantly after adjustment for SO2 (0.2%), CO (0.4%), NO2 (4.1%), and O3 (0.4%). Notably, the hazard ratio for GF with the same increase in PM10 concentration increased by 6.2%, and the hazard ratio for mortality increased by 6.8% after adjustment for O3 (Table 4). As a result of subgroup analysis of two groups according to the BPR mechanism, the hazard ratio of PM10 was 1.053 (95% CI: 1.043–2.895) in the TCMR group, and the HR in the ABMR group was 1.133 (95%CI:1.054–1.219), which were statistically significant (Table 5).

**Table 3:** Risk of outcomes of 1-year average PM_10_ concentrations based on a random-effects model

	BPR				DCGF				All-cause mortality			
Variables	HR	95% CI		*P* Value	HR	95% CI		*P* Value	HR	95% CI		*P* Value
Age at transplantation	1	0.991	1.009	0.9275	0.974	0.948	1.002	0.0647	**1.07**	**1.033**	**1.108**	**0.0002**
Smoking	1.215	0.914	1.614	0.1806	1.091	0.507	2.350	0.8235	0.463	0.131	1.631	0.2304
Body mass index	1	0.999	1.001	0.5951	0.928	0.844	1.022	0.1279	0.904	0.800	1.022	0.1077
Hypertension	0.909	0.647	1.277	0.582	1.198	0.274	5.245	0.8108	0.93	0.117	7.397	0.9455
Diabetes mellitus	0.888	0.686	1.148	0.3631	0.895	0.420	1.907	0.7731	1.159	0.499	2.692	0.7314
Sex (male)	1.064	0.870	1.302	0.5473	**2.232**	**1.213**	**4.107**	**0.0098**	1.747	0.821	3.716	0.1477
HLA mismatch	**1.217**	**1.149**	**1.289**	**<.0001**	**1.182**	**1.006**	**1.389**	**0.0419**	1	0.808	1.237	0.9988
Cause of ESRD (DM)	1.158	0.823	1.630	0.3998	2.404	0.958	6.036	0.0617	1.417	0.528	3.804	0.4893
Cause of ESRD (HTN)	1	0.615	1.626	0.9993	1.663	0.471	5.868	0.429	1.369	0.375	4.997	0.6347
Cause of ESRD (GN)	0.955	0.765	1.193	0.6881	0.682	0.374	1.243	0.2113	0.602	0.255	1.420	0.2465
Cause of ESRD (others)	1.018	0.708	1.464	0.9218				^a^				^a^
Phosphorus	1.051	0.992	1.113	0.0918	**1.252**	**1.089**	**1.440**	**0.0016**	1.204	0.997	1.454	0.0535
Albumin	**0.881**	**0.777**	**0.998**	**0.0473**	**0.444**	**0.323**	**0.611**	**<.0001**	0.67	0.440	1.021	0.0622
eGFR at 6 months	**0.987**	**0.981**	**0.993**	**<.0001**	**0.965**	**0.946**	**0.984**	**0.0003**	0.999	0.977	1.022	0.9519
preemptive	0.883	0.696	1.120	0.3042	0.815	0.445	1.494	0.509	0.999	0.440	2.266	0.9978
Annual mean PM_10_	**1.058**	**1.047**	**1.068**	**<.0001**	**1.049**	**1.014**	**1.084**	**0.0055**	**1.093**	**1.046**	**1.142**	**<.0001**
Donor age	**1.012**	**1.004**	**1.020**	**0.0039**	0.993	0.971	1.016	0.5561	1.005	0.978	1.033	0.7132
Donor sex	1.16	0.954	1.410	0.137	1.066	0.633	1.795	0.8099	1.537	0.760	3.112	0.2319
Donor type (Deceased)	**1.365**	**1.080**	**1.725**	**0.0091**	1.724	0.884	3.361	0.1098	1.939	0.890	4.223	0.0955
ABO-incompatible	**2.002**	**1.374**	**2.918**	**0.0003**	1.897	0.549	6.558	0.3117	1.183	0.148	9.467	0.8743
Random	Covariance estimates(θ)			p-value	Covariance estimates(θ)			p-value	Covariance estimates(θ)			p-value
regions	0.05304			0.0078	0.0001			0.0128	0.03079			0.1866

Data are presented as mean and standard deviation unless otherwise indicated. Covariates are given as measured at the time of kidney transplantation. *BPR* biopsy-proven rejection, *DCGF* death-censored graft failure, *HLA* human leukocyte antigen, *ESRD* end stage renal disease, *DM* diabetes mellitus, *HTN* hypertension, *GN* glomerulonephritis, *eGFR* estimated glomerular filtration rate. The multivariable model was adjusted for age, smoking, BMI, hypertension, DM, sex, HLA mismatch, cause of ESRD, serum phosphorus, albumin, eGFR at 6 months, preemptive, annual mean PM_10_, donor age, sex, donor type, ABO-incompatible and random effect (region)

^a^These are unestimable due to the lack of the events

**Table 4:** Hazard Ratio in association with annual average concentration of PM10 after adjusting for co-pollutants

	BPR			DCGF			All-cause mortality		
	HR	95%CI		HR	95%CI		HR	95%CI	
Single pollutant model of PM10	1.053	1.042	1.063	1.049	1.014	1.084	1.09	1.043	1.140
PM10 with adjustment for SO2 (sulfur dioxide)	1.051	1.040	1.062	1.046	1.011	1.081	1.084	1.037	1.133
Single pollutant model of PM10	1.053	1.042	1.063	1.049	1.014	1.084	1.09	1.043	1.140
PM10 with adjustment for CO (carbon monoxide)	1.049	1.038	1.059	1.037	1.001	1.073	1.075	1.027	1.125
Single pollutant model of PM10	1.053	1.042	1.063	1.049	1.014	1.084	1.09	1.043	1.140
PM10 with adjustment for NO2 (Nitrogen dioxide)	1.012	1.004	1.020	1.048	1.013	1.084	1.09	1.042	1.139
Single pollutant model of PM10	1.053	1.042	1.063	1.049	1.014	1.084	1.09	1.043	1.140
PM10 with adjustment for O3 (Ozone)	1.049	1.039	1.060	1.111	1.071	1.152	1.142	1.086	1.201

*BPR* biopsy-proven rejection, *DCGF* death-censored graft failure

Adjusted covariates: age, smoking, BMI, hypertension, DM, sex, HLA mismatch, cause of ESRD, serum phosphorus, albumin, eGFR at 6 months, preemptive, annual mean PM_10_, donor age, sex, donor type, and ABO-incompatible

**Table 5:** The results of subgroup analysis by types of BPR

	TCMR				ABMR			
Variables	HR	95% CI		P Value	HR	95% CI		P Value
Age at transplantation	0.998	0.989	2.739	0.6959	**1.073**	**1.016**	**1.134**	**0.0117**
Smoking	1.148	0.846	4.277	0.3769	3.067	0.535	17.590	0.2085
Body mass index	1	0.995	2.730	0.8204	0.986	0.843	1.154	0.8641
Hypertension	0.999	0.675	4.017	0.9963	0.624	0.102	3.803	0.6089
Diabetes mellitus	0.884	0.675	3.171	0.3698	0.569	0.142	2.282	0.4263
Sex (male)	1.152	0.929	3.922	0.1963	0.441	0.138	1.406	0.1661
HLA mismatch	**1.219**	**1.147**	**3.596**	**<.0001**	1.19	0.843	1.680	0.3219
Cause of ESRD (DM)	1.153	0.803	4.552	0.44	0.826	0.108	6.312	0.8539
Cause of ESRD (HTN)	1.065	0.652	4.738	0.8025				^a^
Cause of ESRD (GN)	0.945	0.745	3.263	0.6416	1.682	0.486	5.818	0.4116
Cause of ESRD (others)	1.032	0.706	4.104	0.8693	0.406	0.036	4.564	0.4652
Phosphorus	1.042	0.981	3.010	0.1828	0.995	0.686	1.442	0.9773
Albumin	0.906	0.791	2.835	0.1528	0.652	0.328	1.297	0.2231
eGFR at 6 months	**0.989**	**0.982**	**2.707**	**0.0012**	1.001	0.962	1.041	0.9796
preemptive	0.869	0.672	3.083	0.2849	1.062	0.329	3.432	0.9196
Annual mean PM_10_	**1.053**	**1.043**	**2.895**	**<.0001**	**1.133**	**1.054**	**1.219**	**0.0008**
Donor age	**1.014**	**1.006**	**2.780**	**0.0009**	0.998	0.953	1.044	0.9208
Donor sex	1.13	0.918	3.808	0.2491	2.051	0.604	6.957	0.2497
Donor type (Deceased)	**1.348**	**1.057**	**4.913**	**0.0162**	0.525	0.261	4.241	0.9435
ABO-incompatible	**1.179**	**0.701**	**5.474**	**0.5346**	**5.89**	**1.184**	**29.297**	**0.0303**

Data are presented as mean and standard deviation unless otherwise indicated. Covariates are given as measured at the time of kidney transplantation. *TCMR* acute T-cell mediated rejection, *ABMR* acute antibody-mediated rejection, *DCGF* death-censored graft failure, *HLA* human leukocyte antigen, *ESRD* end stage renal disease, *DM* diabetes mellitus, *HTN* hypertension, *GN* glomerulonephritis, *eGFR* estimated glomerular filtration rate. The multivariable model was adjusted for age, smoking, BMI, hypertension, DM, sex, HLA mismatch, cause of ESRD, serum phosphorus, albumin, eGFR at 6 months, preemptive, annual mean PM_10_, donor age, sex, donor type, and ABO-incompatible

^a^hese are unestimable due to the lack of the events

## Discussion

To the best of our knowledge, this is the first study to examine the association between long-term exposure to PM10 and clinical outcomes in KTRs. In this study, we demonstrated that long-term exposure to higher 1-year moving mean PM10 concentration significantly increased the risk of all-cause mortality, DCGF, and BPR in KTRs. We used the observations of the monitoring stations at the local level and applied the average values over several periods to conduct a sensitivity analysis. In addition, by considering the unmeasured confounder, the characteristic of regions, we used models with a random effect term. When considering the within-cluster correlation of survival times, random effects were significant only in DCGF and BPR and not mortality. The results of both models, the model with random effects or not, showed that PM10 had a statistically significant effect on the health of kidney transplant patients.

Several epidemiological studies have reported the association between particulate matter and CKD. For example, a weak acceleration in the progression of albuminuria was observed during chronic exposure to PM10 in the Multi-Ethnic Study of Atherosclerosis cohort. In China, long-term exposure to PM2.5 was associated with an increased risk of membranous nephropathy in 71,151 native kidney biopsy samples [14]. Another longitudinal observational cohort study of US veterans reported the association between PM2.5 concentration and a higher risk of CKD incidence and progression to ESRD [8]. Among 21,656 Taiwanese adults [15], individual exposure to PM10, coarse particles, or PM2.5 was related to reduced renal function. In a nationwide cohort study [9], Bowe et al. showed that higher concentrations of PM10, NO_2_, and CO, were associated with increased risks of CKD, renal function decline, and ESRD. In a recent cohort study in the US observed that higher annual PM2.5 exposure was associated the increased albuminuria and a higher risk of CKD [16].

In the present study, we found that elevated levels of PM10 were consistently associated with a significantly higher risk of adverse renal transplantation outcomes, including DCGF, BPR, and all-cause mortality. Our sensitivity analyses indicated that our results were robust, including the examination of various exposure durations and analyses involving fully adjusted models. We found that PM10 affects each outcome even after adjusting for other air pollutants. In addition, the effects of CO and O3, which are other air pollutants that were adjusted, were statistically significant.

Table 3 shows the covariance estimates and associated statistics for fixed effects and the variance of frailty. As shown in the table, the frailty value is significantly greater than zero (θ = 0.05; *p* value = 0.0078). Furthermore, our result shows that there are factors that influnence the hazard of BPR. It is obvious from our results, that the districts were important (p value = 0.0078) and gamma frailty with a covariance estimates of 0.05 shows that there were unmeasured districts effects present in the model.

The underlying biological mechanisms that may explain the novel association between long-term PM10 exposure and renal outcomes in KTRs is unclear. Classically, the inhalation of particulate matters activates pulmonary inflammatory cells that may trigger a systemic inflammatory response that triggers a cascade of events, ultimately affecting the body’s cardiovascular system [17]. Recent studies have reported that the inhaled nanoparticles rapidly translocate from the pulmonary tissues into the vascular systemic circulation and subsequently accumulate in various organs, such as the heart, liver, and kidneys; these observations have been made in studies involving both animals and humans [18–21]. These particles have the capacity to induce pulmonary inflammation and oxidative stress; parallel pathways can also be activated in the systemic circulation and organ systems. Research has also shown that inflammatory markers, including C-reactive protein, fibrinogen, white blood cell counts, and IL-6, are positively associated with PM exposure [22, 23], and that short-term changes in PM concentrations cause alterations in these key inflammatory biomarkers [24, 25].

Other studies have investigated kidney damage following sub-chronic exposure to PM2.5 by analyzing the levels of early kidney biomarkers, histological changes, induction of the angiotensin and bradykinin system, and by measuring changes in blood pressure [26, 27]. In a previous study, involving an animal model, long-term exposure to PM2.5 was associated with kidney damage, including inflammatory cell infiltration, tubulo-interstitial fibrosis, and mesangial expansion; collectively, these effects caused an impairment in renal function [28].

Our data showed that prolonged long-term exposure to PM10 was associated with higher risks of transplant kidney rejections in KTRs. However, there is little in the current literature to explain the relationship between PM10 exposure and the risk of graft rejections. However, there is some evidence originating from studies involving the recipients of other transplanted organs, such as lung transplant recipients that suggests that PM may trigger rejection of the transplanted organ. Recent studies have reported that exposure to traffic-related air pollution independently increased the risk of bronchiolitis obliterans syndrome, a condition that is clinically correlated with chronic rejection in lung transplant patients [29], and also associated with the development of chronic lung allograft dysfunction, a condition that is related to both acute and chronic allograft rejections [30, 31]. Acute rejection presents a major problem after organ transplantation, and is a recognized risk factor for chronic rejection and all-cause mortality. The authors of these previous papers provided evidence for the increased risk of rejection after lung transplantation with temporal changes in particulate air pollution, and reported this was associated with bronchoalveolar lavage neutrophilia and lymphocytosis. This tissue damage was explained by the novel effect of PM on Th17 polarization via the aryl hydrocarbon receptor [32]. However, the precise biological mechanisms by which exposure to PM10 can trigger kidney rejection is not yet fully understood. One explanation is that the inhaled PM may directly or indirectly induce oxidative stress and inflammation in the transplanted kidney and that this induces endothelial cell dysfunction and exacerbates the production of reactive oxygen species. These complicated processes may be responsible for shifting the immune responses to an effector phenotype, thus leading to the loss of self-tolerance and a shift towards allograft rejection. Further preclinical and epidemiological studies are now required to investigate the association between air pollution and allograft rejection, especially in KTRs.

One particular strength of our study is that we investigated a large contemporary Korean cohort of KTRs with a long-term follow-up of 15 years. Because we had access to comprehensive long-term air pollutant data, we were able to adjust the data for multiple donors and recipient variables. Moreover, this study used BPR data as the chief outcome to evaluate the impact of PM10. However, this study also has several limitations that need to be considered. First, the study participants were mostly South Koreans; therefore, the findings may not be generalizable to other populations. Although we accounted for known confounding factors, and region with frailty survival analysis, we cannot ignore the possibility and potential effects of unknown or non-measured confounders. Therefore, if the sample size of the study was large, and subjects were evenly distributed in the sub-regional unit, it can be carefully assumed that the effect of size on the region may increase.

## Conclusions

In summary, we demonstrate a significant association between PM10 concentrations and the risk of graft failure development, all-cause mortality, and BPR in KTRs. Continued efforts to improve air quality may help to reduce the burden of renal outcome in KTRs.

## Supplementary Information

**Additional file 1: Figure S1.** Location map of the study participants. **Table S1.** Risk of BPR in PM 10 concentration by exposure durations from events day. **Table S2.** Risk of DCGF in PM 10 concentration by exposure durations from events day. **Table S3.**. Risk of all-cause mortality in PM 10 concentration by exposure durations from events day. **Table S4.** Association between average annual PM10 and SO2, CO, NO2 and O3 exposure in outcomes after kidney transplant.

## Data Availability

The datasets generated during and/or analyzed during the current study are available from the corresponding author on reasonable request.

## Abbreviations

PM10: 10-μm particulate matter
KTRs: Kidney transplant recipients
DCGF: Death-censored graft failure
TCMR: Acute T-cell mediated rejection
ABMR: Acute antibody-mediated rejection
BPR: Biopsy-proven rejection
CKD: Chronic kidney disease
eGFR: Estimated glomerular filtration rate
BMI: Body mass index
RRT: Renal replacement therapy
SD: Standard deviation
HR: Hazard ratio
CI: Confidence interval
