# Supplementary material for: Clinical outcomes associated with long-term exposure to airborne particulate pollution in kidney transplant recipients
Source: Environ Health. 2021 May 15;20:61. doi: 10.1186/s12940-021-00741-y (PMC8126074; doi:10.1186/s12940-021-00741-y)
Supplement: Supplementary file 1 — Additional file 1: Figure S1. Location map of the study participants. Table S1. Risk of BPR in PM 10 concentration by exposure durations from events day. Table S2. Risk of DCGF in PM 10 concentration by exposure durations from events day. Table S3.. Risk of all-cause mortality in PM 10 concentration by exposure durations from events day. Table S4. Association between average annual PM10 and SO2, CO, NO2 and O3 exposure in outcomes after kidney transplant. [file 12940_2021_741_MOESM1_ESM.docx]

**Supplementary Information**

*For*

**Clinical outcomes associated with long-term exposure to airborne particulate pollution in kidney transplant recipients**

**Figure S1. Location map of the study participants.**

**Table S1. Risk of BPR in PM 10 concentration by exposure durations from events day**

**Table S2. Risk of DCGF in PM 10 concentration by exposure durations from events day**

**Table S3. Risk of all-cause mortality in PM 10 concentration by exposure durations from events day**

**Table S4. Association between average annual PM10 and SO2, CO, NO2 and O3 exposure in outcomes after kidney transplant.**

**Figure S1. Location map of the study participants.**

**Table S1. Risk of BPR in PM 10 concentration by exposure durations from events day**

| Exposure duration(year) | 2 | | | 3 | | | 4 | | | 5 | | |
| --- | --- | --- | --- | --- | --- | --- | --- | --- | --- | --- | --- | --- |
| Variables | HR | 95%CI | | HR | 95%CI | | HR | 95%CI | | HR | 95%CI | |
| Age at transplantation | 1 | 0.991 | 1.009 | 1 | 0.991 | 1.009 | 1 | 0.992 | 1.009 | 1 | 0.992 | 1.009 |
| Smoking | 1.16 | 0.875 | 1.539 | 1.15 | 0.867 | 1.526 | 1.149 | 0.866 | 1.525 | 1.139 | 0.858 | 1.511 |
| Body mass index | 1 | 0.999 | 1.001 | 1 | 0.999 | 1.001 | 1 | 0.999 | 1.001 | 1 | 0.999 | 1.001 |
| Hypertension | 0.908 | 0.648 | 1.274 | 0.882 | 0.628 | 1.238 | 0.836 | 0.595 | 1.174 | 0.817 | 0.581 | 1.148 |
| Diabetes mellitus | 0.846 | 0.655 | 1.092 | 0.852 | 0.660 | 1.101 | 0.875 | 0.677 | 1.130 | 0.889 | 0.688 | 1.149 |
| Sex | 1.081 | 0.884 | 1.322 | 1.088 | 0.890 | 1.331 | 1.051 | 0.859 | 1.286 | 1.043 | 0.852 | 1.276 |
| HLA mismatch | 1.218 | 1.150 | 1.291 | 1.214 | 1.146 | 1.286 | 1.201 | 1.134 | 1.273 | 1.197 | 1.129 | 1.268 |
| Cause of ESRD (DM) | 1.163 | 0.826 | 1.637 | 1.165 | 0.827 | 1.640 | 1.185 | 0.841 | 1.671 | 1.177 | 0.835 | 1.659 |
| Cause of ESRD (HTN) | 0.98 | 0.604 | 1.591 | 0.973 | 0.600 | 1.579 | 1.028 | 0.633 | 1.668 | 1.04 | 0.641 | 1.688 |
| Cause of ESRD (GN) | 0.958 | 0.769 | 1.194 | 0.959 | 0.769 | 1.195 | 0.978 | 0.785 | 1.219 | 0.972 | 0.780 | 1.212 |
| Cause of ESRD (others) | 0.982 | 0.684 | 1.408 | 0.987 | 0.688 | 1.415 | 1.002 | 0.698 | 1.439 | 0.993 | 0.691 | 1.426 |
| Phosphorus | 1.05 | 0.991 | 1.111 | 1.05 | 0.992 | 1.112 | 1.049 | 0.991 | 1.110 | 1.049 | 0.991 | 1.111 |
| Albumin | 0.903 | 0.797 | 1.022 | 0.912 | 0.806 | 1.032 | 0.91 | 0.804 | 1.030 | 0.904 | 0.799 | 1.023 |
| eGFR at 6 months | 0.987 | 0.981 | 0.993 | 0.988 | 0.981 | 0.994 | 0.989 | 0.982 | 0.995 | 0.989 | 0.983 | 0.996 |
| preemptive | 0.884 | 0.698 | 1.119 | 0.886 | 0.700 | 1.123 | 0.909 | 0.717 | 1.153 | 0.917 | 0.723 | 1.163 |
| Annual mean PM10 | 1.049 | 1.039 | 1.060 | 1.049 | 1.039 | 1.060 | 1.056 | 1.046 | 1.066 | 1.061 | 1.051 | 1.071 |
| Donor age | 1.012 | 1.004 | 1.020 | 1.012 | 1.004 | 1.020 | 1.014 | 1.006 | 1.022 | 1.015 | 1.006 | 1.023 |
| Donor sex | 1.146 | 0.944 | 1.390 | 1.151 | 0.949 | 1.397 | 1.143 | 0.942 | 1.387 | 1.149 | 0.946 | 1.395 |
| Donor type(Deceased) | 1.35 | 1.071 | 1.702 | 1.346 | 1.068 | 1.696 | 1.345 | 1.067 | 1.695 | 1.327 | 1.053 | 1.673 |
| ABO-incompatible | 1.926 | 1.325 | 2.798 | 1.951 | 1.342 | 2.837 | 1.981 | 1.362 | 2.883 | 1.988 | 1.366 | 2.894 |

Data are presented as mean with standard deviation unless otherwise indicated. Covariates as measured at the time of kidney transplantation. BPR, biopsy-proved rejection; HLA, human leukocyte antigen; ESRD, end stage renal disease; DM, diabetes mellitus; HTN, hypertension; GN, glomerulonephritis; eGFR, estimated glomerular filtration rate.

**Table S2. Risk of DCGF in PM 10 concentration by exposure durations from events day**

| Exposure duration(year) | | 2 | | | 3 | | | | 4 | | | | 5 | | | |  |
| --- | --- | --- | --- | --- | --- | --- | --- | --- | --- | --- | --- | --- | --- | --- | --- | --- | --- |
| Variables | HR | | 95%CI | | | HR | 95%CI | | | HR | 95%CI | | | HR | 95%CI | | |
| Age at transplantation | 0.978 | | 0.952 | 1.005 | | 0.978 | 0.952 | 1.004 | | 0.983 | 0.957 | 1.010 | | 0.979 | 0.954 | 1.006 | |
| Smoking | 1.138 | | 0.531 | 2.438 | | 1.113 | 0.518 | 2.392 | | 1.148 | 0.529 | 2.494 | | 1.109 | 0.513 | 2.395 | |
| Body mass index | 0.934 | | 0.850 | 1.026 | | 0.933 | 0.849 | 1.025 | | 0.92 | 0.834 | 1.014 | | 0.934 | 0.848 | 1.029 | |
| Hypertension | 1.193 | | 0.273 | 5.216 | | 1.153 | 0.263 | 5.053 | | 1 | 0.226 | 4.426 | | 0.962 | 0.217 | 4.252 | |
| Diabetes mellitus | 0.843 | | 0.397 | 1.792 | | 0.845 | 0.398 | 1.796 | | 0.875 | 0.410 | 1.868 | | 0.85 | 0.398 | 1.814 | |
| Sex | 2.096 | | 1.155 | 3.804 | | 2.107 | 1.161 | 3.822 | | 2.051 | 1.112 | 3.785 | | 1.985 | 1.091 | 3.612 | |
| HLA mismatch | 1.178 | | 1.005 | 1.381 | | 1.178 | 1.004 | 1.381 | | 1.16 | 0.987 | 1.365 | | 1.17 | 0.998 | 1.371 | |
| Cause of ESRD (DM) | 2.303 | | 0.922 | 5.754 | | 2.294 | 0.917 | 5.738 | | 2.269 | 0.896 | 5.743 | | 2.227 | 0.881 | 5.625 | |
| Cause of ESRD (HTN) | 1.52 | | 0.434 | 5.321 | | 1.531 | 0.438 | 5.354 | | 1.691 | 0.481 | 5.942 | | 1.568 | 0.450 | 5.469 | |
| Cause of ESRD (GN) | 0.657 | | 0.362 | 1.193 | | 0.66 | 0.364 | 1.197 | | 0.702 | 0.383 | 1.287 | | 0.647 | 0.356 | 1.176 | |
| Cause of ESRD (others) | * | |  |  | | * |  |  | | * |  |  | | * |  |  | |
| Phosphorus | 1.257 | | 1.095 | 1.443 | | 1.258 | 1.096 | 1.444 | | 1.259 | 1.095 | 1.447 | | 1.245 | 1.085 | 1.428 | |
| Albumin | 0.454 | | 0.331 | 0.622 | | 0.453 | 0.331 | 0.620 | | 0.452 | 0.328 | 0.625 | | 0.448 | 0.328 | 0.612 | |
| eGFR at 6 months | 0.965 | | 0.946 | 0.984 | | 0.965 | 0.947 | 0.984 | | 0.967 | 0.948 | 0.986 | | 0.968 | 0.949 | 0.987 | |
| preemptive | 0.781 | | 0.429 | 1.423 | | 0.785 | 0.431 | 1.432 | | 0.823 | 0.448 | 1.509 | | 0.811 | 0.443 | 1.486 | |
| Annual mean PM10 | 1.05 | | 1.012 | 1.089 | | 1.061 | 1.022 | 1.102 | | 1.098 | 1.058 | 1.139 | | 1.103 | 1.065 | 1.142 | |
| Donor age | 0.992 | | 0.970 | 1.014 | | 0.991 | 0.970 | 1.014 | | 0.993 | 0.971 | 1.016 | | 0.992 | 0.970 | 1.014 | |
| Donor sex | 1.106 | | 0.660 | 1.854 | | 1.125 | 0.670 | 1.891 | | 1.228 | 0.717 | 2.104 | | 1.213 | 0.714 | 2.061 | |
| Donor type (Deceased) | 1.61 | | 0.833 | 3.110 | | 1.607 | 0.833 | 3.100 | | 1.345 | 0.687 | 2.634 | | 1.546 | 0.809 | 2.953 | |
| ABO-incompatible | 1.86 | | 0.540 | 6.405 | | 1.924 | 0.558 | 6.642 | | 1.989 | 0.571 | 6.928 | | 2.201 | 0.634 | 7.643 | |

Data are presented as mean with standard deviation unless otherwise indicated. Covariates as measured at the time of kidney transplantation. DCGF, death-censored graft failure; HLA, human leukocyte antigen; ESRD, end stage renal disease; DM, diabetes mellitus; HTN, hypertension; GN, glomerulonephritis; eGFR, estimated glomerular filtration rate.

* These were not able to estimate due to the lack of the events.

**Table S3. Risk of all-cause mortality in PM 10 concentration by exposure durations from events day**

| Exposure duration(year) | 2 | | | 3 | | | 4 | | | 5 | | |
| --- | --- | --- | --- | --- | --- | --- | --- | --- | --- | --- | --- | --- |
| Variables | HR | 95%CI | | HR | 95%CI | | HR | 95%CI | | HR | 95%CI | |
| Age at transplantation | 1.072 | 1.036 | 1.110 | 1.068 | 1.031 | 1.106 | 1.068 | 1.032 | 1.105 | 1.069 | 1.033 | 1.107 |
| Smoking | 0.502 | 0.144 | 1.753 | 0.486 | 0.137 | 1.730 | 0.466 | 0.130 | 1.672 | 0.479 | 0.134 | 1.716 |
| Body mass index | 0.916 | 0.813 | 1.031 | 0.897 | 0.793 | 1.013 | 0.916 | 0.811 | 1.034 | 0.919 | 0.812 | 1.040 |
| Hypertension | 1.06 | 0.134 | 8.400 | 1.022 | 0.128 | 8.138 | 0.885 | 0.111 | 7.086 | 0.834 | 0.104 | 6.694 |
| Diabetes mellitus | 1.026 | 0.445 | 2.367 | 1.131 | 0.488 | 2.625 | 1.039 | 0.448 | 2.408 | 1.002 | 0.431 | 2.329 |
| Sex | 1.645 | 0.797 | 3.394 | 1.538 | 0.738 | 3.207 | 1.48 | 0.711 | 3.082 | 1.402 | 0.672 | 2.926 |
| HLA mismatch | 0.995 | 0.809 | 1.223 | 0.999 | 0.808 | 1.235 | 0.994 | 0.804 | 1.229 | 1.003 | 0.811 | 1.240 |
| Cause of ESRD (DM) | 1.424 | 0.539 | 3.760 | 1.519 | 0.575 | 4.010 | 1.631 | 0.613 | 4.338 | 1.748 | 0.651 | 4.696 |
| Cause of ESRD (HTN) | 1.32 | 0.367 | 4.746 | 1.527 | 0.420 | 5.546 | 1.549 | 0.426 | 5.636 | 1.579 | 0.435 | 5.736 |
| Cause of ESRD (GN) | 0.602 | 0.260 | 1.394 | 0.568 | 0.237 | 1.364 | 0.654 | 0.281 | 1.519 | 0.657 | 0.282 | 1.528 |
| Cause of ESRD (others) | * |  |  | * |  |  | * |  |  | * |  |  |
| Phosphorus | 1.231 | 1.024 | 1.479 | 1.243 | 1.033 | 1.497 | 1.197 | 0.992 | 1.444 | 1.185 | 0.982 | 1.429 |
| Albumin | 0.644 | 0.427 | 0.972 | 0.64 | 0.427 | 0.961 | 0.674 | 0.452 | 1.005 | 0.68 | 0.459 | 1.007 |
| eGFR at 6 months | 0.997 | 0.975 | 1.020 | 0.994 | 0.972 | 1.016 | 0.998 | 0.976 | 1.019 | 0.999 | 0.978 | 1.021 |
| preemptive | 0.864 | 0.388 | 1.926 | 0.994 | 0.439 | 2.252 | 0.913 | 0.403 | 2.066 | 0.938 | 0.411 | 2.138 |
| Annual mean PM10 | 1.076 | 1.023 | 1.131 | 1.1 | 1.044 | 1.159 | 1.13 | 1.074 | 1.189 | 1.145 | 1.089 | 1.203 |
| Donor age | 1.002 | 0.975 | 1.029 | 1.004 | 0.977 | 1.031 | 1.005 | 0.979 | 1.032 | 1.007 | 0.980 | 1.035 |
| Donor sex | 1.58 | 0.790 | 3.159 | 1.772 | 0.866 | 3.625 | 1.662 | 0.815 | 3.388 | 1.674 | 0.816 | 3.432 |
| Donor type (Deceased) | 1.749 | 0.824 | 3.708 | 1.809 | 0.834 | 3.924 | 1.708 | 0.799 | 3.652 | 1.671 | 0.781 | 3.577 |
| ABO-incompatible | 1.133 | 0.143 | 8.982 | 1.147 | 0.144 | 9.130 | 1.256 | 0.157 | 10.026 | 1.309 | 0.164 | 10.472 |

Data are presented as mean with standard deviation unless otherwise indicated. Covariates as measured at the time of kidney transplantation. HLA, human leukocyte antigen; ESRD, end stage renal disease; DM, diabetes mellitus; HTN, hypertension; GN, glomerulonephritis; eGFR, estimated glomerular filtration rate.

* These were not able to estimate due to the lack of the events.

**Table S4. Association between average annual PM10 and SO2, CO, NO2 and O3 exposure in outcomes after kidney transplant.**

| Exposure air pollutants | SO2 | | | CO | | | NO2 | | | O3 | | |
| --- | --- | --- | --- | --- | --- | --- | --- | --- | --- | --- | --- | --- |
|  | HR | 95%CI | | HR | 95%CI | | HR | 95%CI | | HR | 95%CI | |
| **Biopsy-proved rejections (BPR)** |  |  |  |  |  |  |  |  |  |  |  |  |
| Age at transplantation | 1 | 0.991 | 1.009 | 1 | 0.991 | 1.009 | 1 | 0.991 | 1.009 | 0.998 | 0.989 | 1.007 |
| Smoking | 1.161 | 0.868 | 1.552 | 1.161 | 0.869 | 1.552 | 1.16 | 0.868 | 1.551 | 1.122 | 0.840 | 1.499 |
| Body mass index | 1 | 0.999 | 1.001 | 1 | 0.999 | 1.001 | 1 | 0.999 | 1.001 | 1 | 0.999 | 1.001 |
| Hypertension | 0.9 | 0.640 | 1.265 | 0.896 | 0.638 | 1.258 | 0.887 | 0.630 | 1.249 | 0.833 | 0.593 | 1.169 |
| Diabetes mellitus | 0.864 | 0.668 | 1.118 | 0.874 | 0.675 | 1.131 | 0.875 | 0.677 | 1.130 | 0.889 | 0.688 | 1.149 |
| Sex | 1.081 | 0.885 | 1.321 | 1.088 | 0.891 | 1.330 | 1.087 | 0.890 | 1.327 | 1.082 | 0.886 | 1.322 |
| HLA mismatch | **1.212** | **1.144** | **1.284** | **1.214** | **1.145** | **1.286** | **1.213** | **1.146** | **1.285** | **1.199** | **1.133** | **1.270** |
| Cause of ESRD (DM) | 1.156 | 0.821 | 1.628 | 1.136 | 0.807 | 1.600 | 1.146 | 0.814 | 1.612 | 1.133 | 0.806 | 1.593 |
| Cause of ESRD (HTN) | 0.993 | 0.611 | 1.613 | 1.027 | 0.631 | 1.671 | 0.976 | 0.601 | 1.585 | 0.954 | 0.587 | 1.552 |
| Cause of ESRD (GN) | 0.967 | 0.773 | 1.208 | 0.968 | 0.774 | 1.210 | 0.961 | 0.769 | 1.201 | 0.924 | 0.740 | 1.154 |
| Cause of ESRD (others) | 0.984 | 0.685 | 1.412 | 0.971 | 0.676 | 1.394 | 0.995 | 0.693 | 1.428 | 0.881 | 0.613 | 1.265 |
| Phosphorus | 1.045 | 0.987 | 1.107 | 1.046 | 0.988 | 1.107 | 1.043 | 0.985 | 1.105 | 1.025 | 0.967 | 1.087 |
| Albumin | **0.879** | **0.776** | **0.997** | 0.882 | 0.778 | 1.000 | 0.885 | 0.781 | 1.003 | 0.884 | 0.780 | 1.001 |
| eGFR at 6 months | **0.987** | **0.981** | **0.994** | **0.988** | **0.981** | **0.994** | **0.987** | **0.981** | **0.994** | **0.987** | **0.981** | **0.994** |
| preemptive | 0.901 | 0.710 | 1.142 | 0.887 | 0.700 | 1.125 | 0.886 | 0.699 | 1.123 | 0.926 | 0.730 | 1.175 |
| Annual mean air pollutant | 1.056 | 0.982 | 1.136 | **1.002** | **1.001** | **1.003** | 0.997 | 0.987 | 1.007 | **1.02** | **1.016** | **1.024** |
| Annual mean PM10(㎍/㎥) | **1.051** | **1.040** | **1.062** | **1.049** | **1.038** | **1.059** | **1.053** | **1.043** | **1.063** | **1.049** | **1.039** | **1.060** |
| Donor age | **1.013** | **1.005** | **1.021** | **1.012** | **1.004** | **1.020** | **1.012** | **1.004** | **1.020** | **1.015** | **1.006** | **1.023** |
| Donor sex | 1.143 | 0.940 | 1.389 | 1.138 | 0.937 | 1.383 | 1.148 | 0.945 | 1.395 | **1.228** | **1.010** | **1.494** |
| Donor type(Deceased) | **1.322** | **1.046** | **1.671** | **1.309** | **1.036** | **1.654** | **1.362** | **1.078** | **1.720** | **1.345** | **1.065** | **1.698** |
| ABO-incompatible | **1.852** | **1.266** | **2.707** | **1.91** | **1.306** | **2.792** | **1.926** | **1.324** | **2.803** | **1.853** | **1.275** | **2.691** |
| **Graft failure** |  |  |  |  |  |  |  |  |  |  |  |  |
| Age at transplantation | 0.973 | 0.946 | 1.001 | 0.973 | 0.946 | 1.000 | 0.974 | 0.947 | 1.001 | **0.961** | **0.933** | **0.991** |
| Smoking | 1.439 | 0.759 | 2.730 | 1.475 | 0.782 | 2.782 | 1.495 | 0.791 | 2.825 | 1.652 | 0.781 | 3.493 |
| Body mass index | 0.929 | 0.844 | 1.022 | 0.928 | 0.843 | 1.022 | 0.924 | 0.839 | 1.017 | 0.906 | 0.807 | 1.017 |
| Hypertension | 1.18 | 0.269 | 5.173 | 1.258 | 0.287 | 5.518 | 1.149 | 0.259 | 5.090 | 0.374 | 0.083 | 1.686 |
| Diabetes mellitus | 0.942 | 0.439 | 2.023 | 0.928 | 0.436 | 1.973 | 0.888 | 0.416 | 1.893 | 1.013 | 0.458 | 2.237 |
| Sex | **2.108** | **1.137** | **3.908** | **2.1** | **1.132** | **3.897** | **2.087** | **1.126** | **3.868** | **1.939** | **1.009** | **3.725** |
| HLA mismatch | 1.171 | 0.997 | 1.376 | 1.166 | 0.995 | 1.365 | **1.178** | **1.003** | **1.383** | **1.236** | **1.048** | **1.457** |
| Cause of ESRD (DM) | 2.319 | 0.925 | 5.814 | 2.249 | 0.892 | 5.666 | 2.435 | 0.971 | 6.109 | **3.44** | **1.288** | **9.190** |
| Cause of ESRD (HTN) | 1.615 | 0.457 | 5.703 | 1.58 | 0.447 | 5.582 | 1.627 | 0.461 | 5.749 | 3.467 | 0.891 | 13.491 |
| Cause of ESRD (GN) | 0.702 | 0.383 | 1.286 | 0.673 | 0.366 | 1.238 | 0.706 | 0.385 | 1.295 | 0.881 | 0.462 | 1.680 |
| Cause of ESRD (others) | * |  |  | * |  |  | * |  |  | * | - | - |
| Phosphorus | **1.241** | **1.078** | **1.428** | **1.242** | **1.081** | **1.428** | **1.243** | **1.081** | **1.430** | 1.014 | 0.875 | 1.175 |
| Albumin | **0.441** | **0.321** | **0.606** | **0.431** | **0.312** | **0.595** | **0.44** | **0.320** | **0.605** | **0.529** | **0.381** | **0.736** |
| eGFR at 6 months | **0.965** | **0.946** | **0.984** | **0.965** | **0.947** | **0.984** | **0.965** | **0.946** | **0.984** | **0.964** | **0.944** | **0.985** |
| preemptive | 0.785 | 0.424 | 1.451 | 0.76 | 0.413 | 1.400 | 0.772 | 0.418 | 1.425 | 0.61 | 0.319 | 1.164 |
| Annual mean air pollutant | 1.143 | 0.915 | 1.427 | **1.004** | **1.000** | **1.007** | 1 | 0.972 | 1.028 | **1.081** | **1.063** | **1.099** |
| Annual mean PM10 | **1.046** | **1.011** | **1.081** | **1.037** | **1.001** | **1.073** | **1.048** | **1.013** | **1.084** | **1.111** | **1.071** | **1.152** |
| Donor age | 0.993 | 0.971 | 1.016 | 0.996 | 0.973 | 1.019 | 0.994 | 0.971 | 1.016 | 1.005 | 0.981 | 1.031 |
| Donor sex | 1.111 | 0.658 | 1.877 | 1.028 | 0.610 | 1.733 | 1.08 | 0.640 | 1.823 | 1.466 | 0.824 | 2.606 |
| Donor type(Deceased) | 1.64 | 0.839 | 3.208 | 1.627 | 0.834 | 3.172 | 1.637 | 0.838 | 3.199 | 1.475 | 0.704 | 3.089 |
| ABO-incompatible | 1.804 | 0.511 | 6.370 | 1.919 | 0.551 | 6.678 | 1.96 | 0.566 | 6.782 | 5.152 | 1.379 | 19.248 |
| **All-cause mortality** |  |  |  |  |  |  |  |  |  |  |  |  |
| Age at transplantation | **1.073** | **1.035** | **1.112** | **1.073** | **1.035** | **1.113** | **1.073** | **1.036** | **1.112** | **1.062** | **1.017** | **1.110** |
| Smoking | 1.192 | 0.540 | 2.629 | 1.162 | 0.525 | 2.568 | 1.286 | 0.575 | 2.874 | 1.953 | 0.790 | 4.826 |
| Body mass index | 0.92 | 0.818 | 1.035 | 0.916 | 0.813 | 1.032 | 0.914 | 0.812 | 1.028 | 0.88 | 0.752 | 1.031 |
| Hypertension | 0.994 | 0.123 | 8.016 | 1.078 | 0.133 | 8.747 | 0.852 | 0.105 | 6.904 | 0.723 | 0.085 | 6.119 |
| Diabetes mellitus | 1.072 | 0.460 | 2.496 | 1.154 | 0.497 | 2.680 | 1.088 | 0.469 | 2.521 | 1.016 | 0.407 | 2.536 |
| Sex | 1.517 | 0.702 | 3.277 | 1.54 | 0.713 | 3.324 | 1.496 | 0.689 | 3.247 | 1.556 | 0.659 | 3.674 |
| HLA mismatch | 0.994 | 0.806 | 1.226 | 1.01 | 0.819 | 1.246 | 0.989 | 0.801 | 1.220 | 1.099 | 0.883 | 1.368 |
| Cause of ESRD (DM) | 1.286 | 0.483 | 3.420 | 1.24 | 0.462 | 3.326 | 1.346 | 0.504 | 3.596 | 2.219 | 0.718 | 6.856 |
| Cause of ESRD (HTN) | 1.389 | 0.383 | 5.032 | 1.369 | 0.378 | 4.961 | 1.442 | 0.395 | 5.260 | 1.289 | 0.275 | 6.036 |
| Cause of ESRD (GN) | 0.629 | 0.267 | 1.484 | 0.613 | 0.258 | 1.455 | 0.631 | 0.267 | 1.494 | 0.779 | 0.325 | 1.871 |
| Cause of ESRD (others) | * |  |  | * |  |  | * |  |  | * |  |  |
| Phosphorus | **1.214** | **1.003** | **1.471** | 1.206 | 0.998 | 1.457 | 1.199 | 0.990 | 1.453 | 0.98 | 0.808 | 1.189 |
| Albumin | 0.65 | 0.428 | 0.987 | 0.652 | 0.428 | 0.992 | 0.65 | 0.430 | 0.984 | 0.742 | 0.510 | 1.081 |
| eGFR at 6 months | 1 | 0.977 | 1.022 | 1 | 0.978 | 1.023 | 0.999 | 0.977 | 1.022 | 0.997 | 0.976 | 1.018 |
| preemptive | 0.998 | 0.440 | 2.266 | 1.01 | 0.448 | 2.275 | 0.93 | 0.410 | 2.109 | 0.876 | 0.356 | 2.158 |
| Annual mean air pollutant | 1.204 | 0.891 | 1.628 | **1.004** | **1.000** | **1.009** | 0.986 | 0.952 | 1.021 | **1.075** | **1.056** | **1.094** |
| Annual mean PM10 | **1.084** | **1.037** | **1.133** | **1.075** | **1.027** | **1.125** | **1.09** | **1.042** | **1.139** | **1.142** | **1.086** | **1.201** |
| Donor age | 1.004 | 0.977 | 1.032 | 1.003 | 0.976 | 1.031 | 1.006 | 0.979 | 1.034 | 1.003 | 0.973 | 1.034 |
| Donor sex | 1.667 | 0.829 | 3.350 | 1.561 | 0.775 | 3.144 | 1.681 | 0.831 | 3.402 | 1.419 | 0.661 | 3.043 |
| Donor type(Deceased) | 1.723 | 0.806 | 3.686 | 1.736 | 0.817 | 3.689 | 1.723 | 0.804 | 3.693 | 1.647 | 0.685 | 3.958 |
| ABO-incompatible | 1.264 | 0.155 | 10.296 | 1.276 | 0.157 | 10.358 | 1.277 | 0.157 | 10.357 | 2.533 | 0.258 | 24.882 |

Data are presented as mean with standard deviation unless otherwise indicated. Covariates as measured at the time of kidney transplantation. HLA, human leukocyte antigen; ESRD, end stage renal disease; DM, diabetes mellitus; HTN, hypertension; GN, glomerulonephritis; eGFR, estimated glomerular filtration rate.

* These were not able to estimate due to the lack of the events.
